# Potential Use of School Absenteeism Record for Disease Surveillance in Developing Countries, Case Study in Rural Cambodia

**DOI:** 10.1371/journal.pone.0076859

**Published:** 2013-10-14

**Authors:** Calvin K. Y. Cheng, Hing Channarith, Benjamin J. Cowling

**Affiliations:** 1 School of Public Health, The University of Hong Kong, Pokfulam, Hong Kong Special Administrative Region, China; 2 The Cambodian Children’s Advocacy Foundation, Phnom Penh, Cambodia; Arizona State University, United States of America

## Abstract

**Background:**

Disease surveillance allows prospective monitoring of patterns in disease incidence in the general community, specific institutions (e.g. hospitals, elderly care homes), and other important population subgroups. Surveillance activities are now routinely conducted in many developed countries and in certain easy-to-reach areas of the developing ones. However due to limited health resources, population in rural area that consisted of the most the vulnerable groups are not under surveillance. Cheaper alternative ways for disease surveillance were needed in resource-limited settings.

**Methods and Findings:**

In this study, a syndromic surveillance system using disease specific absenteeism rates was established in 47 pre-schools with 1,417 students 3–6 y of age in a rural area of Kampot province, Cambodia. School absenteeism data were collected via short message service. Data collected between 1st January and 31st December 2012 was used for system evaluation for future potential use in larger scale. The system appeared to be feasible and acceptable in the rural study setting. Moderate correlation was found between rates of school absenteeism due to illness and the reference data on rates of attendance at health centers in persons <16 y (maximum cross-correlation coefficient = 0.231 at lag = −1 week).

**Conclusions:**

School absenteeism data is pre-existing, easily accessible and requires minimum time and resources after initial development, and our results suggest that this system may be able to provide complementary data for disease surveillance, especially in resource limited settings where there is very little information on illnesses in the community and traditional surveillance systems are difficult to implement. An important next step is to validate the syndromic data with other forms of surveillance including laboratory data.

## Introduction

Disease surveillance provides important information about patterns in diseases in the community [1], [2]. Traditional surveillance systems such as laboratory based surveillance and sentinel surveillance at inpatient and outpatient clinics have been routinely done in many developed countries. However due to limited health resources, surveillance activities can only be held in certain easy-to-reach areas of developing countries [3]. Populations in rural area often have reduced accessibility to health care facilities and thus difficult to monitor. Cheaper alternative approaches to disease surveillance are needed to collect information on illnesses in these communities.

In recent years, newer syndromic surveillance systems have been developed to provide complementary disease information for making public health decisions. These systems include telephone triage [4], over-the-counter pharmaceutical sales [5], internet searches [6], [7] and absenteeism surveillance [8], etc. Most studies of these syndromic surveillance systems focused mainly on detecting early health seeking behaviors before the patients enter the health care facilities. The systems increased the timeliness of outbreak detections by traditional surveillance systems and thus promote situational awareness and, in some cases, timely public health interventions [9], [10], [11]. In addition, syndromic surveillance systems can have other advantages in terms of cost and population coverage.

One of the effective and efficient sources for monitoring disease activity is the use of school absenteeism data [12], [13]. Studies in some developed countries [8], [14], [15], [16], [17] have demonstrated the added value of school absenteeism data for surveillance of influenza-like illnesses (ILI). School absenteeism is potentially a cheaper alternative way for community based disease surveillance. However additional challenges are need to be considered for implementing such surveillance systems in resource limited settings. These include inferior information technology infrastructures, unknown disease transmission patterns, poverty and other reasons for non-illness absenteeism, and lack of capable staff etc. To date, there are very limited studies for studying the feasibility and practical usage of school absenteeism for disease surveillance in developing countries. In this study, we set up a short message service (SMS) based disease surveillance system using school absenteeism data in 47 pre-schools in Bantey Meas and two nearby districts of the Kampot province, in Cambodia and evaluated its performance using data collected in the year 2012.

## Materials and Methods

This study was approved by the institutional review board of the University of Hong Kong. The institutional review board waived the need for written informed consent from the participants.

### Recruitment

We collaborated with the Cambodian Children’s Advocacy Foundation (CCAF), a local Cambodian non-governmental organization for pre-school education to set up the surveillance system. Two types of schools, informal pre-schools run by CCAF and formal pre-schools run by the government were enrolled in this study. Invitation letters were sent electronically to the chief executive director of CCAF to recruit its pre-schools, whereas recruitment of public schools was done by communicating with local education department officials through CCAF. Recruitment began in December 2010. Once the schools agreed to participate, CCAF staff would meet the school staff to explain the study procedures. Standard SMS contact methods were introduced to the school staff for data collection. We also invited health centers that encompassed the areas of the schools in the Bantey Meas district to provide their patients attendance records as reference data for our study, via CCAF’s local connections.

### Data Collection

Attendance records were aggregated by office staff in each school for administrative purposes on a daily basis. Copies of electronic school attendance reports were sent from individual school staff to CCAF staff weekly on Friday by SMS or by direct telecommunication. CCAF staff processed the aggregated data and generated data files in comma separated values (csv) format. The CCAF staff would then compile and send the collected data to the School of Public Health, The University of Hong Kong (HKU) via internet message application protocol for downstream data processing. Data files were standardized including columns of date, school identification number and type, total number of students (including male to female ratio) in each school level and number of school absentees stratified by reasons of absence. The study protocol was approved by the Institutional Review Board of the University of Hong Kong.

For the health centers, a standard record form for the surveillance study was available at the consultation rooms to record two main categories of sickness, namely respiratory and diarrhea cases. When patients attended the clinics, their chief complaint was determined by the clinic doctors or nurses. Once the patients’ chief complaint fell into one or more sickness categories on the form, they were recorded to the most fitted category after consultations by the clinicians. Daily patient attendance record was aggregated by a nurse or a health care assistant at the clinics. Copies of electronic reports were sent from clinic staff to CCAF staff via SMS or by direct telecommunication on Monday every week. Similarly, CCAF staff processed the aggregated data and sent to the HKU server. Collected data included columns of date, health center identification number and type, total number of patients visited, number of serious cases that need referral and number of respiratory, diarrhea and feverish cases stratified by age.

### Data Processing

At CCAF server side, initial data cleaning and aggregation was done using Microsoft Excel. At the HKU server side, we prepared scripts for data cleaning, aggregation, analysis and reports generation. All scripts were executed in R version 2.15.1 (R Development Core Team, Vienna, Austria). Weekly overall absenteeism rates were calculated by the total number of absence-days divided by the total population of students. Data stratified by informal school only, public schools only and illness related absenteeism were also generated for analysis.

### Data Dissemination

**Figure 1:**
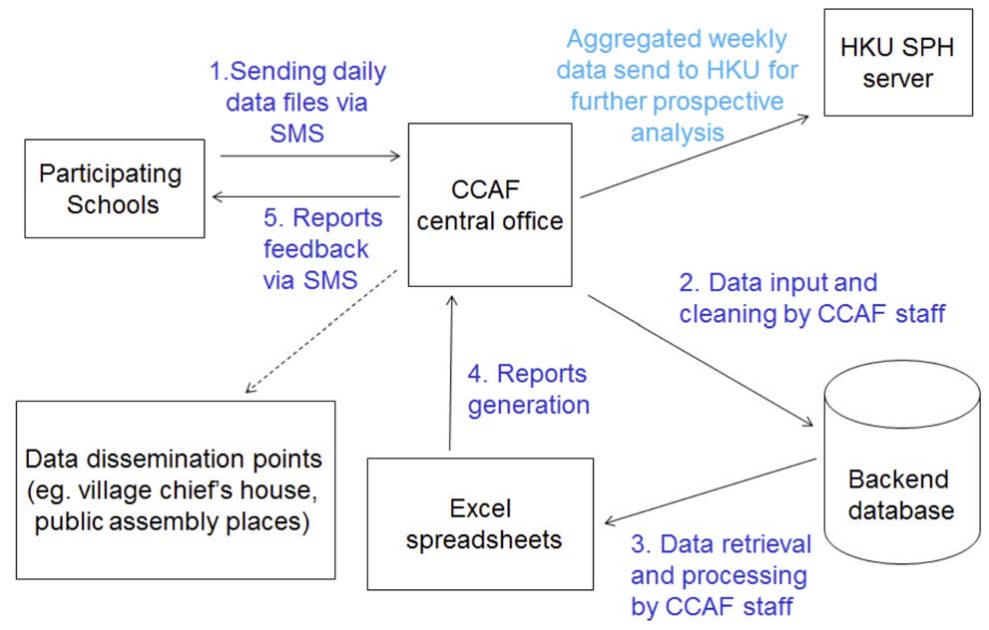
Schematic diagram illustrating data flow of the school absenteeism surveillance system. Broken line arrow indicates the component that will be implemented in future.

Reports of updated absenteeism trend and an interpretation of the overall disease activity in the community were generated and distributed as a feedback to all participating schools through communication between the CCAF and schools staff. Currently we are negotiating with the local officials for a public space to set up a notice board displaying the surveillance results for public reference. The overall system architecture was demonstrated in Figure 1.

### System Evaluation

System performance was evaluated according to United States Centre for disease control and prevention guideline in terms of feasibility, acceptability, data quality and timeliness [18], [19]. Cross-correlation analyses between the absenteeism data and the reference health centers attendance data were done using Pearson’s product-moment correlation. The timeliness of disease peak comparison was assessed by comparing the weeks when the rates in the school data and the reference data were at their highest levels.

## Results

**Figure 2:**
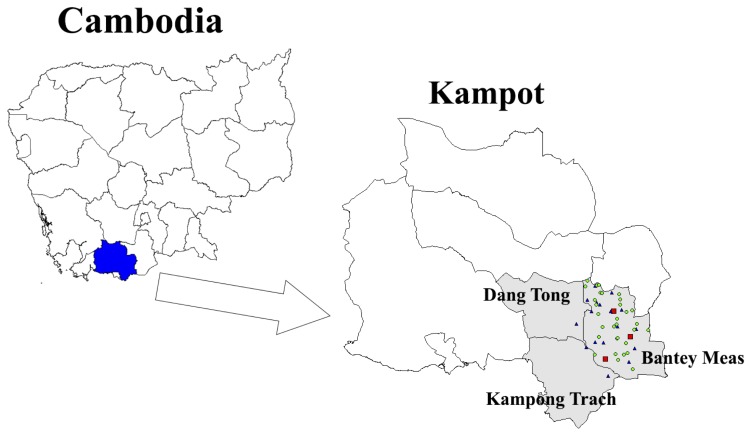
Geographic location of the study sites. Blue triangles indicated the approximate locations of CCAF informal schools, green dots indicated the public schools and red squares indicated the health centers. Most of the study sites were located in the Bantey Meas district.

A total of 336 subjects (with average 47.9% female students) from 17 CCAF informal schools had absenteeism data recorded from 01 December 2010 to 31 December 2012. We obtained the data from 30 public schools with 1,081 students (with average 49.0% female students) from 1 January 2012 to 31 December 2012. Three health centers were under surveillance from 18 February 2012 to 31 December 2012. Nearly all schools (44/47, 93.6%) and all health centers were located in the Bantey Meas district of the Kampot province in Cambodia, while 3 schools were located in 2 nearby districts (Oudong and Kompong Trach district) in the same province (Figure 2). As the study was at the initial stages in year 2011, data quality varied and only the data in 2012 was included for analysis here.

Ongoing communication with CCAF staff revealed that schools and CCAF staff did not have any substantial problems for the data collection procedures except initial issues with the different formats of CCAF and public schools’ data. This problem was resolved in 2011 and the data collection method was acceptable to local staff. The cell phone and SMS network in rural Cambodia did not have data transmission problems throughout the study period. The pilot study period in year 2011 demonstrated that the system implementation was feasible. As the attendance taking procedures were no different from the school staff’s normal practice by adding an extra step for categorizing the absence reasons and a weekly data submission procedure, we did not receive any complaints from the school staff.

Surveillance data were complete in general except for a few weeks where there were transmission delays. The HKU server received an overall of 81% of the participating school data with less than 2 weeks delay. Reasons for delayed data transfer included procedural unfamiliarity for staff, on leave of staff and resending previous missing data after school holidays. On-site data checking in 7 schools on April 2012 and January 2013, including 4 CCAF schools and 3 public schools, revealed that hard-copies of collected data and the submitted electronic data were identical. However, because staff from the local schools could not sometimes classify febrile illnesses into respiratory or other types of illness, the numbers of specific types of illness absenteeism data were quite variable. We used the all cause illness absenteeism in our primary analyses to avoid misclassification and retain data quality.

**Figure 3:**
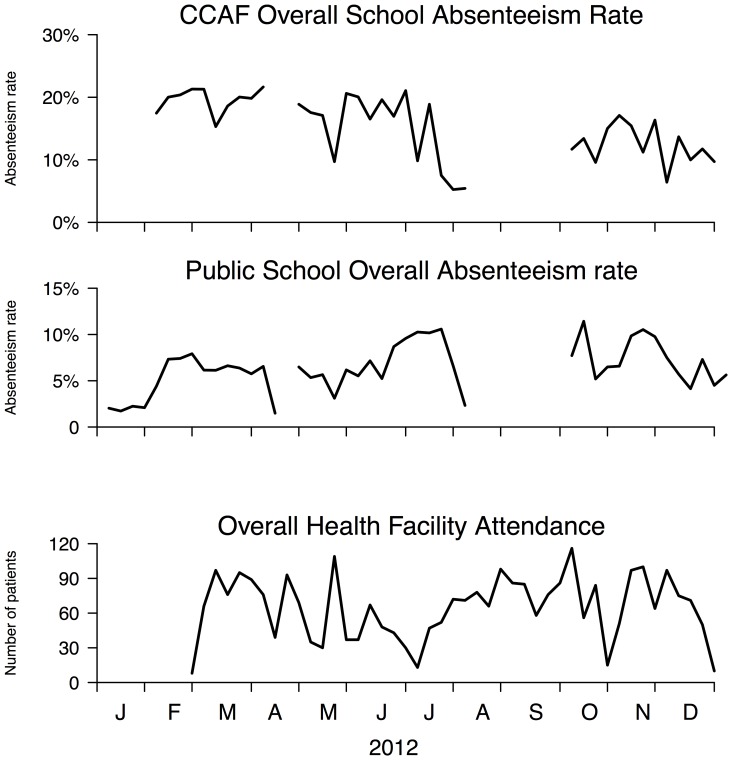
Overall school absenteeism rates compared to the number of all the patients who visited the health centers.

The overall school absenteeism and the reference health center data were shown in Figure 3. Both overall school absenteeism data from formal and informal schools did not have specific patterns or trends, although using public schools data showed less noise than those informal schools empirically, probably due to more regular school attendance requirements. Small sample size (n = 47 with 1,417 students) with non-specific noise may be the main reasons for masking disease patterns, as the average percentage of students absent due to other reasons (eg. no one brings them to school, travel with parents and teacher was absent etc) versus illness specific reasons was considerable (9.7% versus 90.3%).

**Figure 4:**
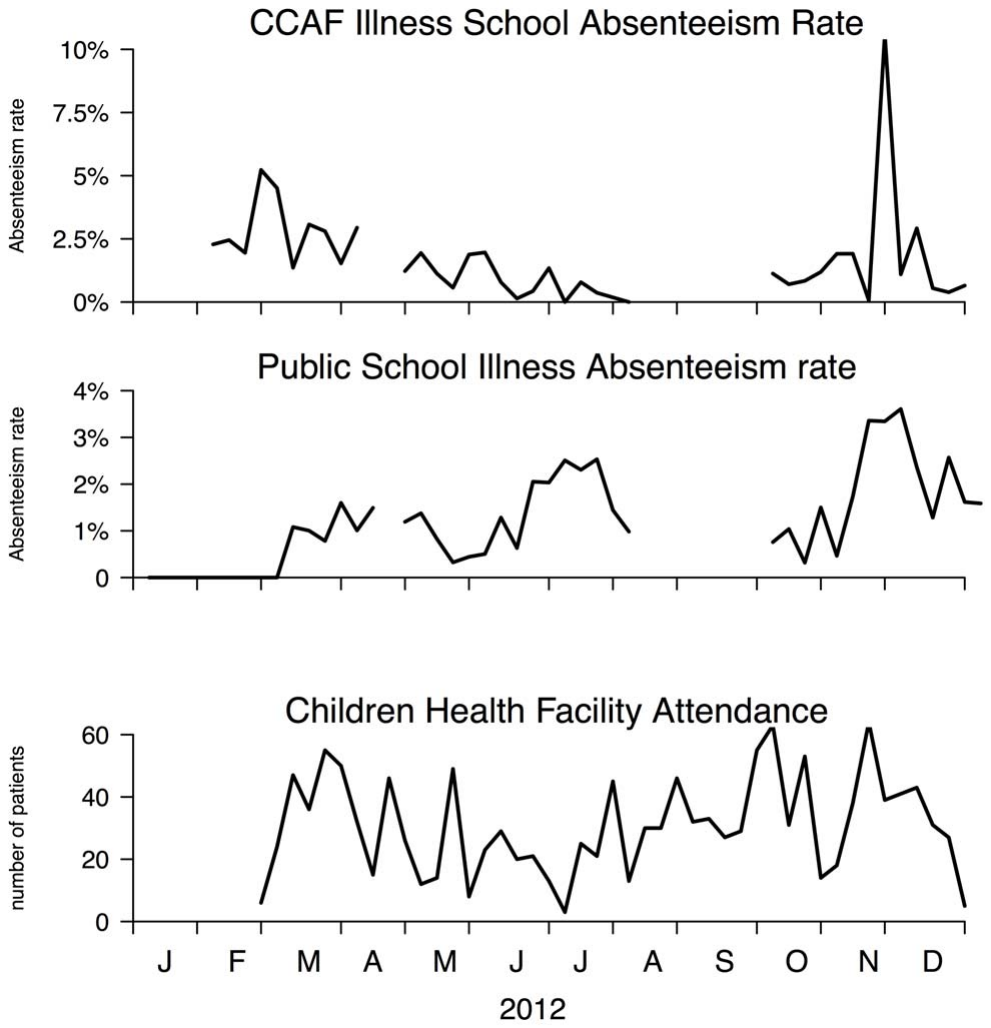
Illness specific school absenteeism rates compared to the number of patients under 16 years old who visited the health centers.

**Figure 5:**
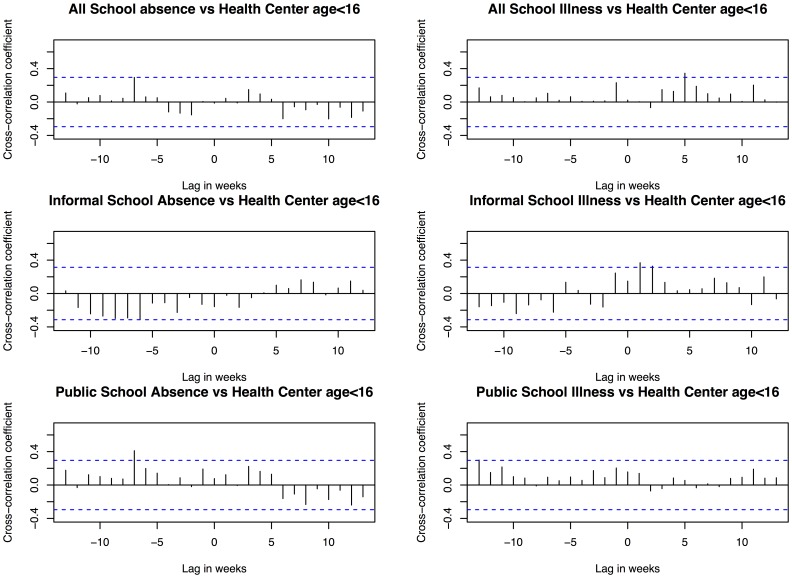
Cross-correlation between the school absenteeism data and the reference health center data. The dotted lines indicated the 95% confidence intervals.

Illness specific absenteeism data revealed that there were apparently two peaks in incidence of illness in 2012. One started in mid-June and another one started in early November. The peaks were sharper using public school data only. We used the health center attendance for children under 16 only as reference to compare with the school absenteeism data. The peak time for school absenteeism leading the reference data was 0.5 weeks on average (Figure 4). Cross-correlation analysis (Figure 5) revealed that there were moderate correlations between illness specific absenteeism and the reference data (maximum cross-correlation coefficient (max ccc) = 0.231 at lag = −1 week, upper 95% CI at 0.295). The correlation increased and was statistically significant when using informal schools data only (max ccc = 0.369 at lag = −1 week, upper 95% CI at 0.314), but decreased when using public school data (max ccc = 0.172 at lag = 1 week, upper 95% CI at 0.295).

## Discussion

The results of our study suggested that the school absenteeism data were feasible and acceptable in rural settings. From the two peaks identified in the illness related school absence data, the one in June-July was suspected to be related to the outbreak of hand, foot and mouth disease caused by enterovirus 71 in Cambodia [20]. The November 2012 peak may be due to a small outbreak of chikungunya infections and is still under investigation.

Because of limited resource and expertise, it is challenging to establish a robust disease surveillance system including laboratory data in settings with limited resources and infrastructure. Sentinel surveillance using outpatient clinic data is also challenging as only very few clinics exist in rural area and often do not have systematic maintenance of medical records. Also the access to care and health seeking behavior of people living in rural area is quite different from those living in the cities for various reasons [21], [22], [23]. These factors tend to hinder the implementation of traditional disease surveillance systems in rural areas. Syndromic surveillance can be feasible, as shown by our study, although the reliability of the information collected in this way remains to be determined.

Sickness absenteeism is well considered to be one of the very first health seeking behaviors for infectious diseases [17]. While school absenteeism data is pre-existing, easily accessible and requires minimum time and resource after initial development, we provided evidence that school absenteeism could be a useful way to capture information on patterns in disease incidence in resource limited settings. In addition, the system also captures those subjects with milder infections who did not seek medical care. While school absenteeism data may have improved coverage and timeliness for these reasons, one of the main disadvantages of such data is the gaps during school holidays. We should also interpret the data with caution as absenteeism can be higher before and after holidays due to holiday effects [24]. While comparing figure 2 and 3, there were other non-illness related absenteeism that masked the true signal of disease activities, such as parents of the students bring them to work together in the city, or the roads to school have been blocked after bad weather. Extra challenges will be encountered to build a useful and effective disease surveillance system in resource limiting settings, for example unreliable staff and undeveloped information technology infrastructure [25].

In this study, using the data from health centers as reference suffered from its small sample size (n = 3, average patients visited per day = 64.8). We tried to compare the surveillance data of those common infectious etiologies of acute febrile illness in Cambodia (eg. influenza, dengue and malaria) [26] with the school data. This coincided with the increases in diseases rates observed during rainy seasons (from May to November) in general. In the future, with the availability of more years of absenteeism data or other surveillance data, it may be possible to explore seasonality more carefully.

In conclusion, in this report we provided evidence that school absenteeism could be a useful approach for disease surveillance in resource limited settings where traditional surveillance systems are difficult to implement. In future we would like to further validate the school data using other sources of available surveillance data, including laboratory testing for biological specimens collected from schools, communities and environment whenever possible. Meanwhile, the system can be improved in future by increasing the degree of automation in data collection, transfer, processing and disseminating, which can further reduce the resources required to maintain the system.
